# Earthquake multi-classification detection based velocity and displacement data filtering using machine learning algorithms

**DOI:** 10.1038/s41598-022-25098-1

**Published:** 2022-12-08

**Authors:** Muhammad Ary Murti, Rio Junior, Ali Najah Ahmed, Ahmed Elshafie

**Affiliations:** 1 Telkom University, Bandung, 40257 Indonesia; 2 Institute of Energy Infrastructure (IEI) and Civil Engineering Department, College of Engineering, Universiti Tenaga Nasional (UNITEN), 43000 Kajang, Selangor Malaysia; 3 Department of Civil Engineering, University of Malaya (UM), 50603 Kuala Lumpur, Malaysia; 4 National Water and Energy Center, United Arab Emirates University, 15551 Al Ain, United Arab Emirates

**Keywords:** Natural hazards, Engineering

## Abstract

Earthquake is one of the natural disasters that have a big impact on society. Currently, there are many studies on earthquake detection. However, the vibrations that were detected by sensors were not only vibrations caused by the earthquake, but also other vibrations. Therefore, this study proposed an earthquake multi-classification detection with machine learning algorithms that can distinguish earthquake and non-earthquake, and vandalism vibration using acceleration seismic waves. In addition, velocity and displacement as integration products of acceleration have been considered additional features to improve the performances of machine learning algorithms. Several machine learning algorithms such as Support Vector Machine (SVM), Random Forest (RF), Decision Tree (DT), and Artificial Neural Network (ANN) have been used to develop the best algorithm for earthquake multi-classification detection. The results of this study indicate that the ANN algorithm is the best algorithm to distinguish between earthquake and non-earthquake, and vandalism vibrations. Moreover, it’s also more resistant to various input features. Furthermore, using velocity and displacement as additional features has been proven to increase the performance of every model.

## Introduction

Indonesia is located between three major tectonic plate confluences, namely the Eurasian Plate, the Indo-Australian Plate, and the Pacific Plate^1^. Based on the records of the Meteorological, Climatological, and Geophysical Agency (BMKG), from 2008–2018 there were around 5000–6000 earthquakes. And in 2019 there have been 15 destructive earthquakes^2^. Earthquake detection using accelerometer sensors has been done by several researchers^3–6^, the vibrations that the sensor will detect are not only vibrations caused by the earthquake, but also other vibrations such as vibrations due to heavy objects dropped on the floor, heavy vehicles passing by, explosions, or when someone is trying to broke the box. Due to similarity of the earthquake waves and those seismic noises, earthquake early warning systems are sometimes accidentally triggered and cause false alert. Therefore, it is necessary to classify earthquake and seismic noise to avoid detection errors^7,8^.

Several researchers have also conducted studies on the use of machine learning in the seismic field. According to Nishita Narvekar^9^, The seismic signal recorded at the earthquake stations are often mixed with noise. Therefore, it is necessary to remove noise before the data is fed to the machine learning algorithm using filtering techniques. In addition, applying Fast Fourier Transform (FFT) as one of the methods that are widely used in the world of Seismology on the seismic signal can be used to reduce computation time^10,11^. Combining it with machine learning algorithms has been proven to give the best results. Furthermore, the experiment of comparison of SVM, DT, and RF algorithms to distinguish between earthquake vibrations and noises shows that RF algorithms give better performance based on this research.

Some researchers^12^, proposed a seismic detection system that can be implemented at the seismic station using ANN and SVM that can classify local earthquakes and the other possibilities vibration. The data were collected from PVAQ station in Portugal. The data is distributed into 60% of training data, 20% of testing data, and 20% of validation data. The performances of the model show that ANN was able to obtain a value greater than 95% whereas SVM is capable to get an almost perfect classification.

In another study^13^, DT is used for solving two classification problems involving signals. The purpose is to learn signal temporal logic (STL) for finding a pattern in data that do not conform to the expected behavior (anomaly detection). The result shows that DT provides good performances and can be interpreted over specific application domains. In another case^14^, DT is applied to classify the conditions of a wind turbine blade by evaluating the turbine vibration signal. There are 600 data samples which 100 samples were from good condition blades. DT classifier that is used for this problem has been proven very much effective for diagnosing this problem. Based on those studies, it shows that the DT algorithm is good for signal and vibration classification.

According to Saman Yaghmaei-Sabegh^15^, characteristics of earthquake ground-motion is an uncertain thing. His study proposed to classify earthquake ground motion using K-means clustering and Self-Organizing Map (SOM) network as two powerful unsupervised clustering techniques by utilizing 6 different scalar frequency content indicators. There are two synthetized and real dataset used in this study. The result shows that T_0_ (The smoothed spectral predominant period) parameter showed the best performances among all scalar indicators. In addition, K-means clustering obtained a better performance than SOM in pattern recognition and classification procedure.

Among various types of machine learning algorithms that have been used by several researchers, recently various studies in a wide range of engineering fields deployed different Machine learning models. Based on their findings the most reliable models are Support Vector Machine (SVM), Random Forest (RF), Decision Tree (DT), and Artificial Neural Network (ANN). Therefore, this study will investigate the reliability of these models for earthquake detection. These models will be used as a comparison to find the best algorithm for earthquake multi-classification detection. The acceleration dataset used is based on seismic events in the Indonesia region, especially on Java Island, recorded at 3 different stations obtained from ESM (Engineering Strong Motion) Database^16^. Accelerometer alone cannot detect shaking pattern of the ground^7^, Hence to overcome this problem, this study proposed to integrate acceleration dataset to obtain velocity and displacement as additional features to improve performances for each algorithm. Therefore, earthquake detection can be done more accurately. The analytical methods used for analyzing machine learning performance in this study will be accuracy, precision, recall, and F1.

## Methodology

### Study area and data collection

The area of this study is Indonesia, focused on Java Island. Java Island is considered the fourth largest island in Indonesia with the highest density of population. It is part of the complex convergence zone between the Eurasian plate and the Indo-Australian plate. Due to that, the Java region witnessed many seismic and volcanic activities. Between 2006 and 2020, earthquakes and other geohazards on volcano-dotted Java Island caused about 7000 deaths, and another 1.8 million people were injured, displaced, or left homeless^17,18^.

**Figure 1:**
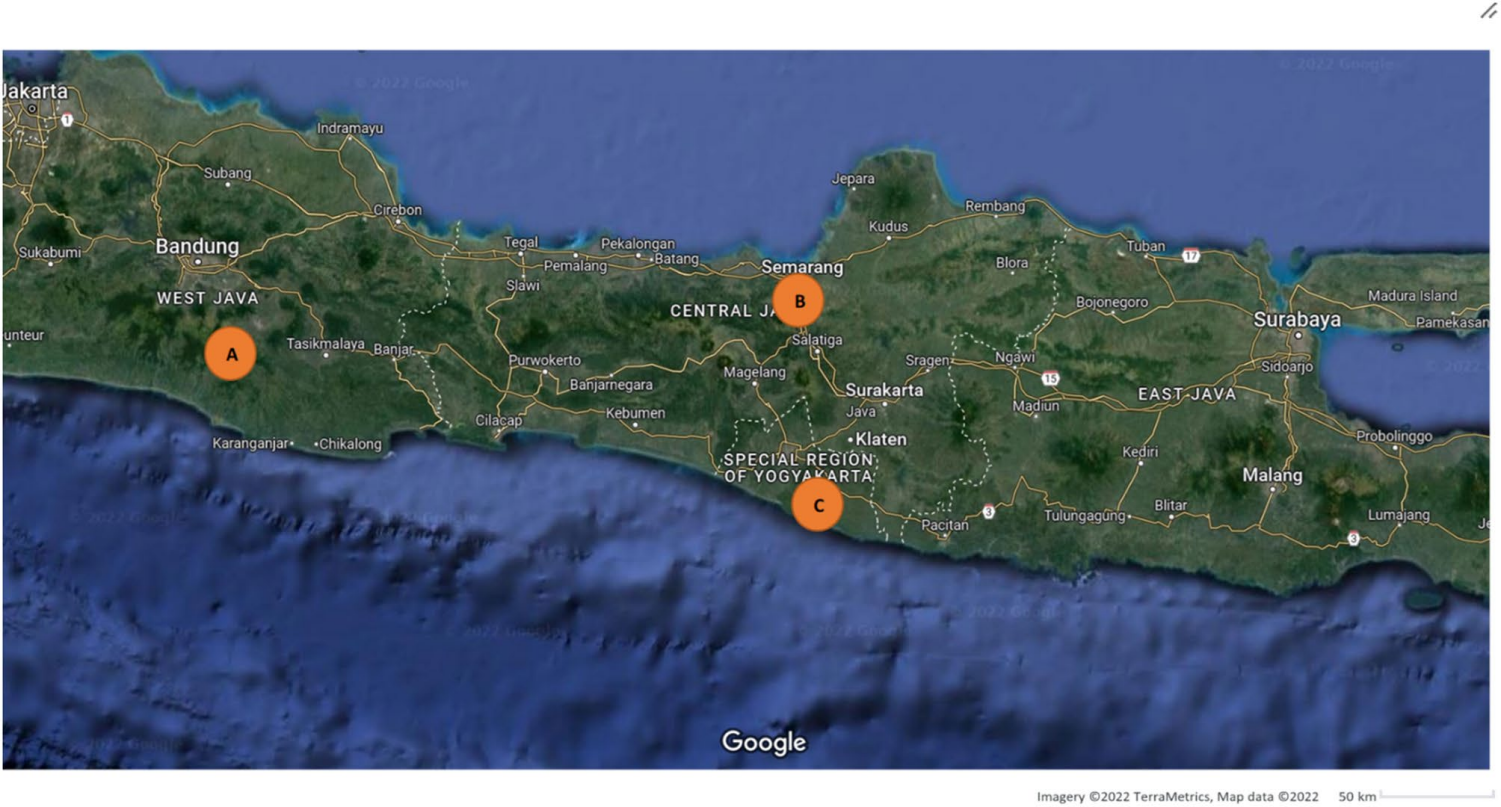
Java Island map with the location of the stations. (**a**). CISI, (**b**). SMRI, (**c**). UGM [Imagery ^©^ 2021 TerraMetrics, Map data^©^ 2022 Google].

Seismic wave acceleration data were collected from ESM Database at 3 different stations, namely CISI, SMRI, and UGM which are located on Java Island as can be seen in Fig. 1.

**Figure 2:**
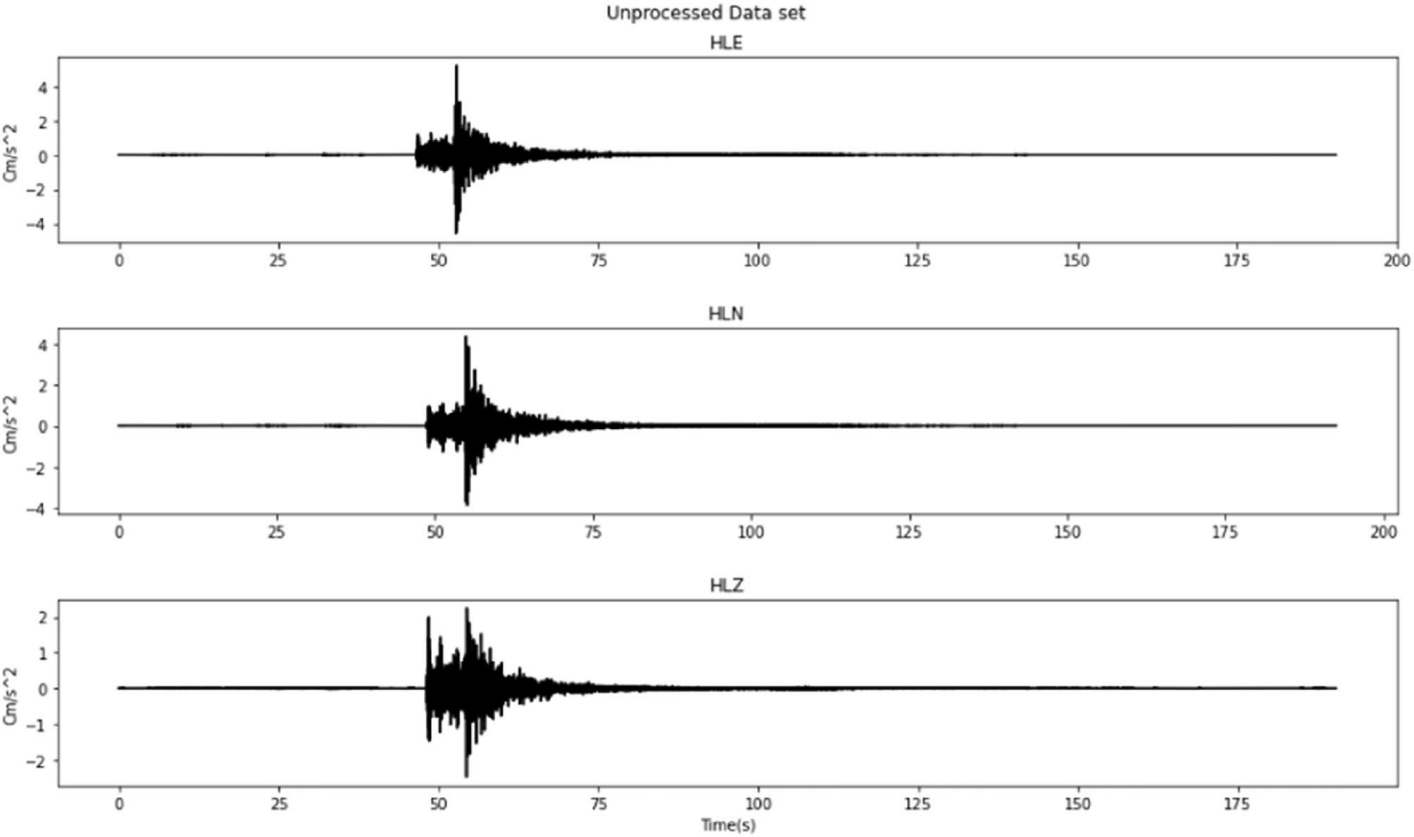
Unprocessed dataset for each channel for 1 event.

**Figure 3:**
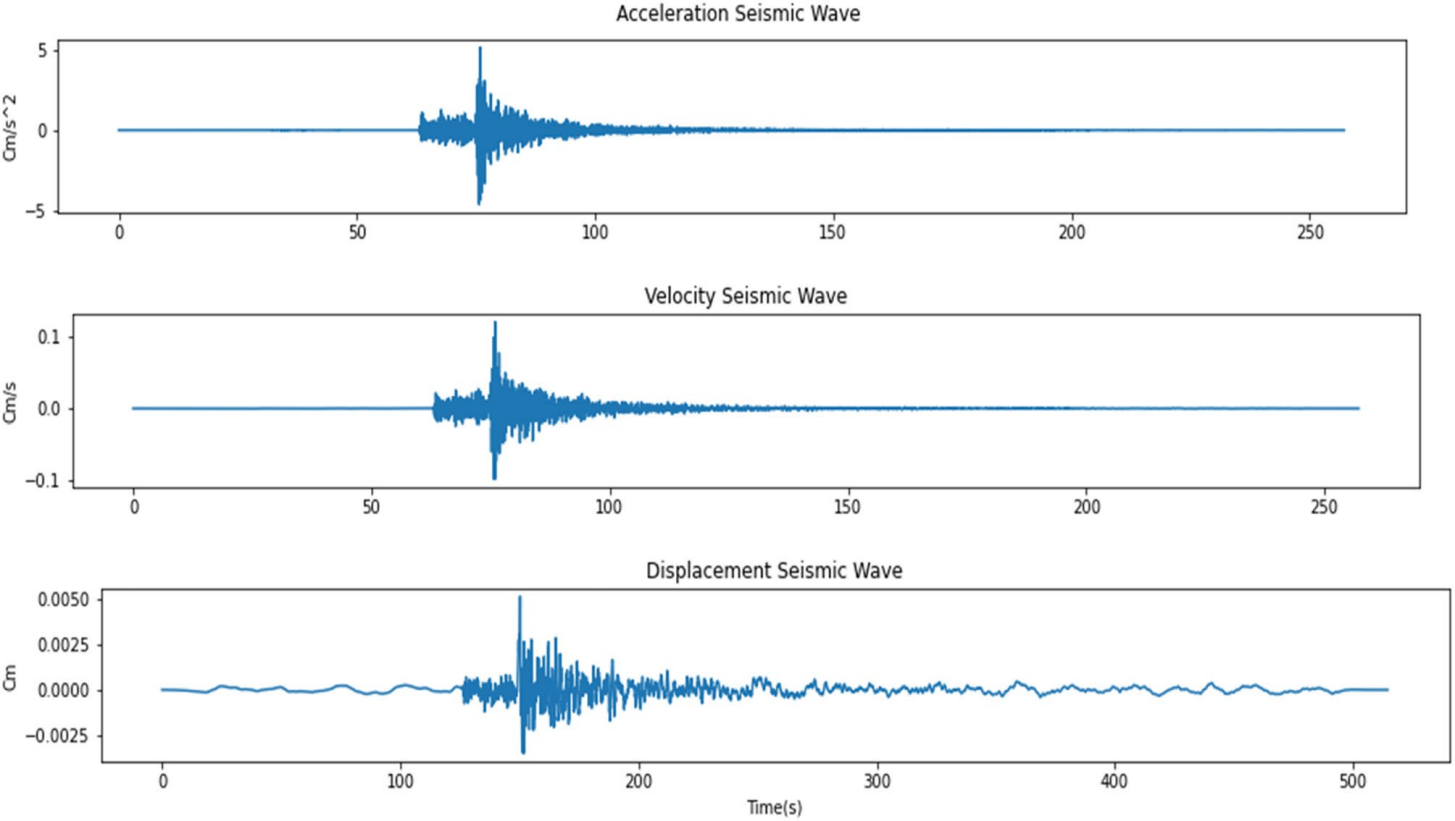
Integration result.

These stations record earthquake events that occurred around Java Island in the past 2006–2009. There are 33 records from CISI, 8 records from SMRI, and 17 records from UGM which make a total of 58 earthquake events that occurred and were recorded around Java Island by those stations. These records contain 3 different channels which are HLE, HLN, and HLZ with acceleration seismic wave information for each channel as shown in Fig. 2. The acceleration seismic wave will be integrated to get velocity and displacement seismic waves which can be used as features to improve models’ performances. Acceleration, velocity, and displacement relation can be described using the math equation^19^:

- Acceleration:
  1 $$Acceleration = a\left( t \right)$$
- Velocity:
  2 $$Velocity \left( {v\left( t \right)} \right) = v_{0} + \mathop \smallint \limits_{0}^{t} a dt$$
- Displacement:
  3 $$Displacement \left( {r\left( t \right)} \right) = r_{0} + \mathop \smallint \limits_{0}^{t} v dt$$
  where, ${v}_{0}$ is the initial value of the velocity and ${r}_{0}$ is the initial position when $t=t-{t}_{0}$. The integration result of the acceleration seismic wave can be seen in Fig. 3.

### Dataset processing

**Figure 4:**
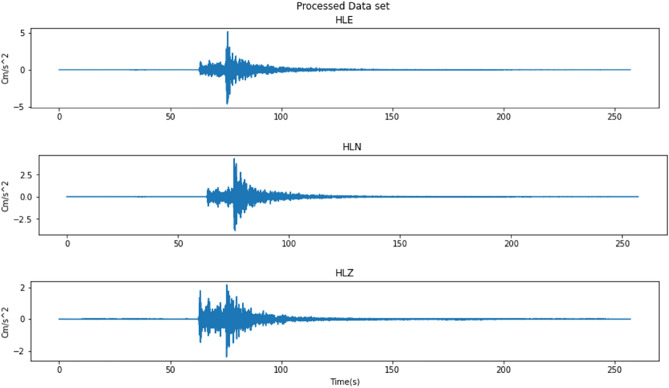
Processed dataset for each channel for 1 event.

Data from ESM Database is in the form of an ASCII file containing detailed event information as well as the acceleration seismic wave data for the event. All the data go through the FFT process to get the frequency domain of the seismic wave and then the frequency is used for the filtering process using a Butterworth Bandpass Filter with the order of filter = 2, minimum frequency = 0.1 Hz, and maximum frequency = 30 Hz to reduce the noises. Figure 4 shows the result of the data filtering process in Fig. 2.

**Figure 5:**
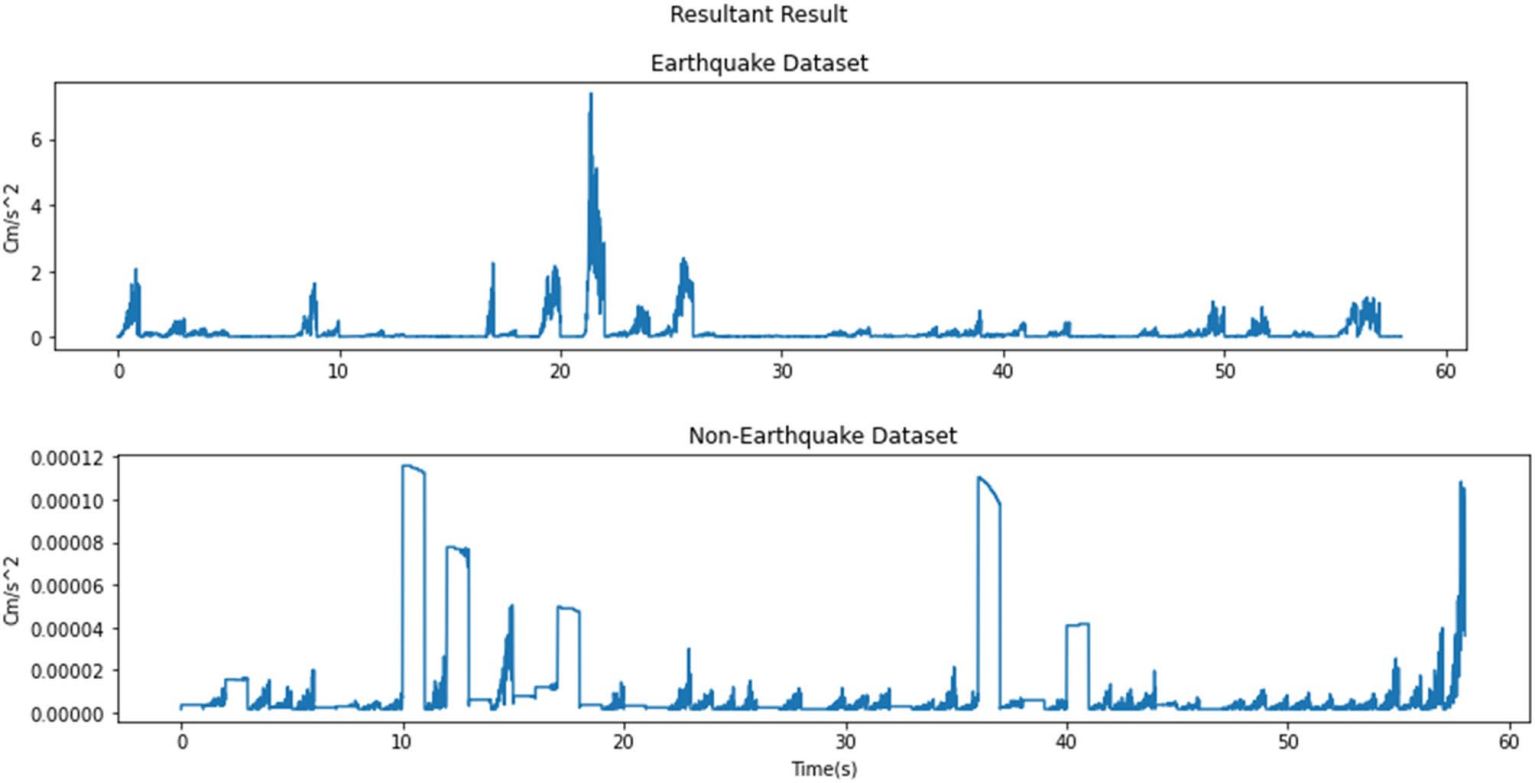
Resultant result.

After the filtering process, the data sampling process will be done. In the data sampling, the acceleration seismic wave data will be split into earthquake and non-earthquake data. The earthquake data and non-earthquake data contain 200 data for 1 seismic event each (equivalent to 1 s because the sampling frequency = 0.005 s) where the earthquake data samples are taken starting from the beginning of P-wave, and the non-earthquake data samples are taken starting from the beginning of the wave until the P-wave arrival. There are a total of 58 seismic events, so each earthquake and non-earthquake dataset will have 3 columns (HLE, HLZ, and HLZ) with 11,600 rows of data for each column [11600, 3]. Next, the 3 columns will be merged into 1 [11600, 1] by using resultant formulas so only amplitude acceleration will be used as a feature. Amplitude resultant result from the total seismic events can be seen in Fig. 5 for both earthquake and non-earthquake. After that, label information will be added to the datasets, 0 represents non-earthquake data and 1 represents earthquake data.

For the vandalism vibration, 2 vandalism datasets were recorded by an accelerometer sensor. Both vandalism datasets will be treated the same as earthquake and non-earthquake datasets. The first vandalism datasets contain 11,600 data and are labeled as 2 which are taken by shaking the table while the sensor is on top of it (made up earthquake). The other datasets contain 750 data labeled as 3 which are taken by the sensor when a heavy vehicle is passing by. There are 4 datasets with a total amount of 35,550 data. The statistical analysis for each dataset is presented in Table 1. Lastly, integration formulas will be applied to all amplitude acceleration datasets to get the amplitude of velocity and displacement which can be used as additional features.

**Table 1:** Statistical analysis of datasets.

Datasets	Features	Mean	Standard deviation	Min	Max	Skewness
Non-earthquake	Acceleration	0.000011	0.000023	0.000002	0.000116	3.387641
	Velocity	5.100214e-06	1.347668e-05	8.660254e-09	1.143856e-04	4.793992
	Displacement	1.655580e-06	5.364661e-06	4.330127e-11	5.763578e-05	6.05555
Earthquake	Acceleration	0.185846	0.463204	0.017488	7.382481	6.367151
	Velocity	0.073343	0.210249	0.000087	2.401821	6.689231
	Displacement	2.022779e-02	6.775113e-02	4.372051e-07	1.070235e + 00	8.752525
Vandalism	Acceleration	0.092205	0.008339	0.055555	0.149810	1.031291
	Velocity	0.046549	0.026731	0.000767	0.103117	0.022305
	Displacement	0.015824	0.014031	0.000008	0.052510	0.641688
Heavy vehicle	Acceleration	0.009519	0.006206	0.000702	0.038378	1.625737
	Velocity	0.025058	0.015861	0.000423	0.068639	0.513051
	Displacement	0.045360	0.042242	0.000085	0.198619	1.016049

### Model selection

Several supervised machine learning algorithms that are used in this study are Support Vector Machine (SVM), Random Forest (RF), Decision Tree (DT), and Artificial Neural Network (ANN). SVM is a supervised learning algorithm that can be used for finding patterns from a complex dataset. SVM is a very powerful and diverse machine learning model, capable of performing linear, non-linear, regression, classification, and outlier detection. When SVM theory was introduced by Vapnik and Cortes in 1995, The SVM was designed for two-group classification (binary classification). The idea behind SVM was previously implemented for the restricted case where the training data can be separated without error. In practice, the SVM has been applied for pattern and digit recognition. This experiment shows SVM can compete with the other classification methods such as decision trees and neural networks^20^. The binary SVM approach can, however, be extended for multiclass scenarios. This will be attained by decomposing the multiclass problem into a series of binary analyses. This can be addressed with a binary SVM by following either the one-against-one or one-against-all strategies^21^.

In SVM,

- input: ${x}_{i}\in {\mathbb{R}}^{D}$, with *D* = feature dimension,
- output: $w$ (weights), one for each feature, whose linear combination produces y (the final output of the SVM model is a decision from the input data).
  4 $$y = w^{T} x_{i} + b$$

with b is bias.

To maximize the margin, the distance from the data points to the hyperplane must be minimized. When the hyperplane cannot separate the two classes perfectly, it is necessary to add a slack variable (${\xi }_{i}$) and hyperparameter C. The function of the hyperparameter is to regulate the use of slack variables, if C is too small, the model can be underfitting, and if C is too large, the model can be overfitting.

5 $$\mathop {{\text{min}}}\limits_{{w,b}} \frac{1}{2}\left\| w \right\|^{2} + C\sum\limits_{{i = 1}}^{m} {\xi _{i} }$$

When the input data cannot be separated linearly, then the data must be mapped to a higher-dimensional space. If the new dimension is very large, it will take a long time to map it. Kernel Tricks can solve this, it works by ostensibly adding features. In this research, the Kernel Gaussian RBF (Radial Basis Functions) will be used.

6 $$K\left( {x,l} \right) = \exp \left( {\left. { - \gamma } \right\|x - \left. l \right\|^{2} } \right)$$

where: *x* = feature vector.

*l* = landmark

$$\gamma = \frac{1}{{2\sigma^{2} }}$$

The DT algorithm can be used for classification and regression. This algorithm can also be used for data with multiple outputs. It performs data classification by forming a tree. Starting from the root node to the leaf node. At each node, there is information on the features that are used as conditions for determining the direction of data flow, gini impurity, the number of samples that arrive at the node, the class prediction value, and the class of the data at that node.

In determining the branch on the DT, information about the gini impurity of the data is needed. Gini impurity evaluates a score in the range between 0 and 1, where 0 is when all observations belong to one class, and 1 is a random distribution of the elements within classes. The feature with the lowest impurity will be selected to be the next branch^22^. In this case, the lower the gini impurity the better the split and the lower the likelihood of misclassification. Equation 7 is gini impurity equation with ${p}_{i}$ is the probability of class *k* at node *i*, and *n* is the number of classes.

7 $${\text{Gini Impurity}}\left( {G_{i} } \right) = 1 - \mathop \sum \limits_{k = 1}^{n} p_{i,k}^{2}$$

RF is an ensemble learning technique consisting of the aggregation of a large number T of decision trees. This technique uses the voting method in determining the classification results. The classification of each DT will be used to determine the final classification. RF uses row and column sampling of the data for each tree. That way each tree is trained using different data. This algorithm can reduce the variance without increasing the bias. In addition, the accuracy of this model can be improved by increasing the CART (ntree) model ensemble^23^.

An artificial neural network (ANN) is an information-processing system that has definite performance characteristics in common with biological neural networks. ANNs are utilized as statistical models in predicting complex systems in engineering. Their enormously parallel structure with a high number of simply connected processing units which are called, neurons—allows the ANN to be utilized for complex, linear as well as non-linear input–output mappings^24–26^.

The most common ANN training method is the backpropagation algorithm. To reduce mistakes, this modifies the weights between neurons. This model is quite effective at identifying patterns. The system can display sluggish convergence and run the danger of a local optimum, but it can rapidly adapt to new data values. A significant challenge is figuring out how many layers there are, how many neurons are in the hidden layer, and how those neurons are connected. The performance of the artificial neural network depends greatly on these factors and issues. Any of these elements could significantly alter the outcomes. For different issues, various ANN architectures will produce various solutions^27^.

All the models will be used to classify earthquake, non-earthquake, and vandalism vibration by training and testing data with a ratio of 70:30. Next, the performances of the models will be determined by analyzing the confusion matrix as one of the common methods used for classification. Table 2 shows the structure of the confusion matrix. From the confusion matrix, some information can be retrieved such as^28,29^:

- Accuracy:
  8 $${\text{Accuracy}} = \frac{TP + TN}{{TP + FP + TN + FN}}$$
- Precision:
  9 $${\text{Precision}} = \frac{TP}{{TP + FP}}$$
- Recall:
  10 $${\text{Recall}} = \frac{TP}{{TP + FN}}$$
- F1:
  11 $$F1 = \frac{TP}{{TP + \frac{1}{2} \left( {FP + FN} \right)}}$$
  Those performances will be used as a comparison for knowing if there is any effect of adding the velocity and displacement as additional features.

**Table 2:** Confusion matrix

	Prediction				
Actual	Labels	0	1	2	3
	0	TN	FP	TN	TN
	1	FN	TP	FN	FN
	2	TN	FP	TN	TN
	3	TN	FP	TN	TN

## Results and discussion

### Analyzing datasets

**Figure 6:**
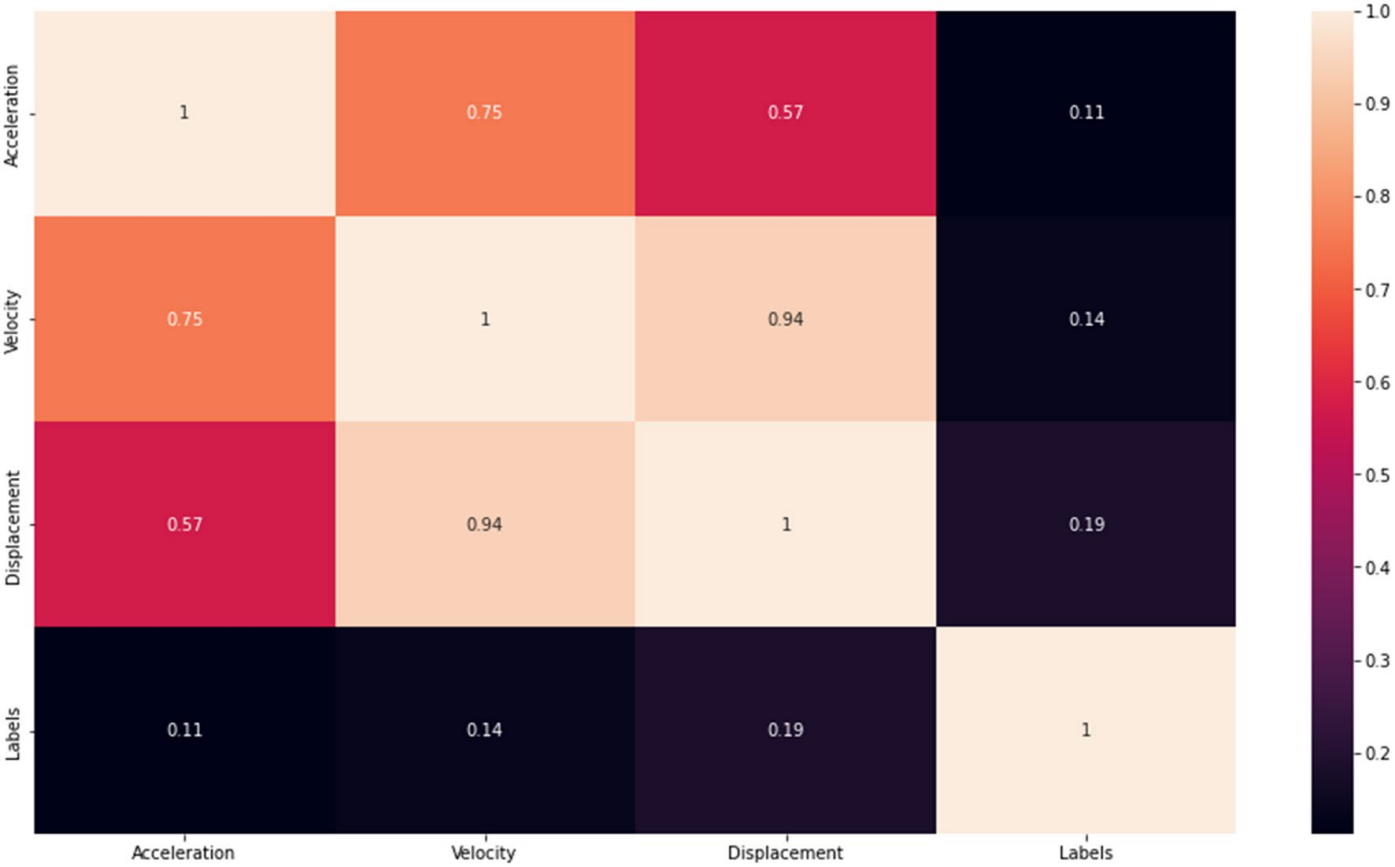
Correlation matrix.

A correlation matrix is a (K x K) square and symmetrical matrix that shows the correlation coefficient between columns i and j of the dataset^30^. Figure 6 shows the correlation matrix between acceleration, velocity, displacement, and labels. The observable result based on Fig. 6 shows acceleration, velocity, and displacement have a fairly close correlation, especially between velocity and displacement which has a value of 0.94 (1 is a perfect linear relationship) meanwhile labels have the least correlation with acceleration, velocity, and displacement.

**Figure 7:**
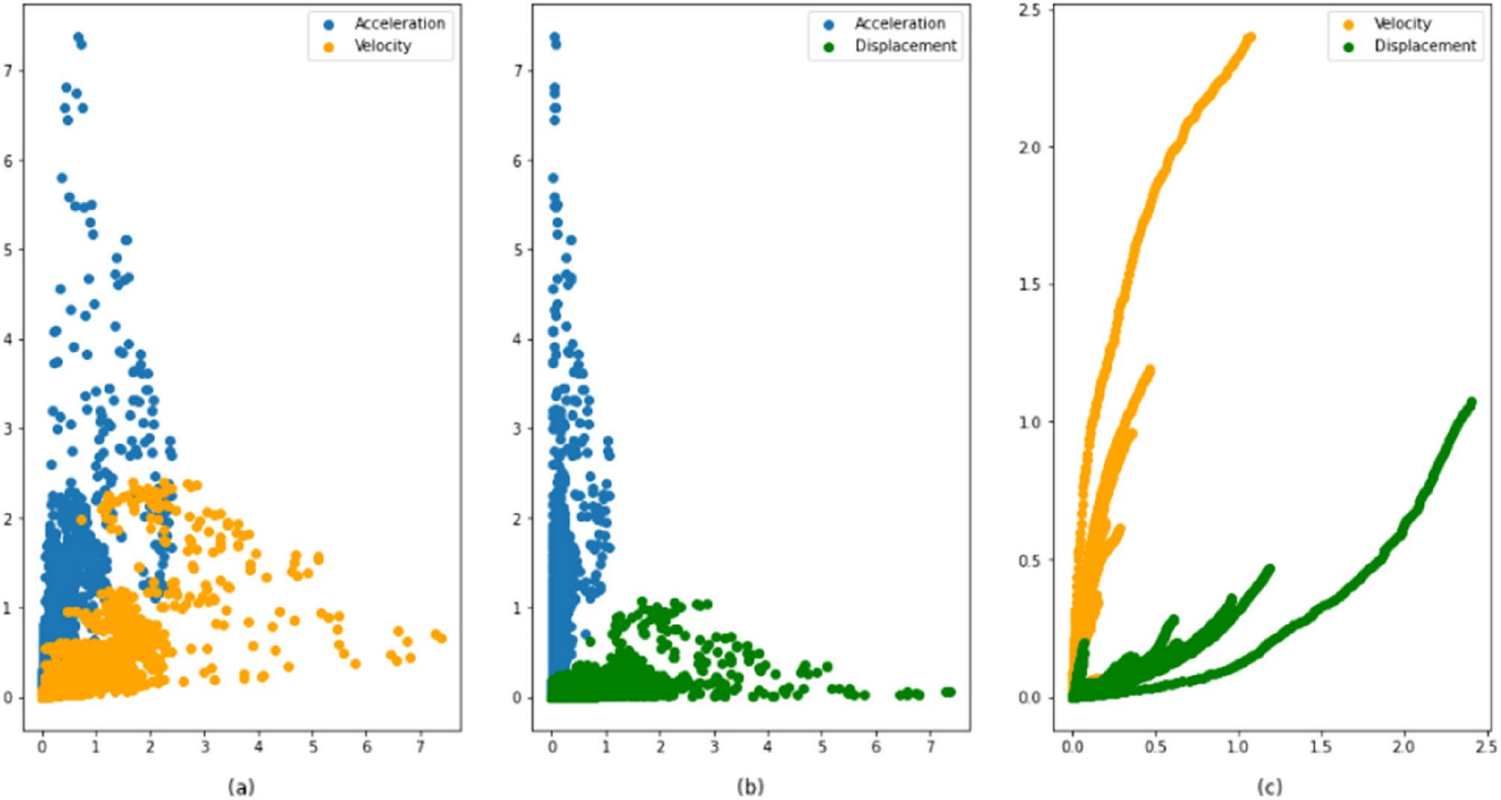
Data distribution. (**a**). Acceleration versus velocity, (**b**) Acceleration versus displacement, (**c**) Velocity versus displacement.

Data distribution is a function that specifies all possible values for a variable and also quantifies the relative frequency (probability of how often they occur). Data distributions are widely used in statistics. Figure 7 shows the data distribution for the dataset. The distribution data for each feature looks good, there is only a small portion of data that is included in isolated data that make the dataset ready to feed to the machine learning algorithms.

### Machine learning algorithm test results

**Figure 8:**
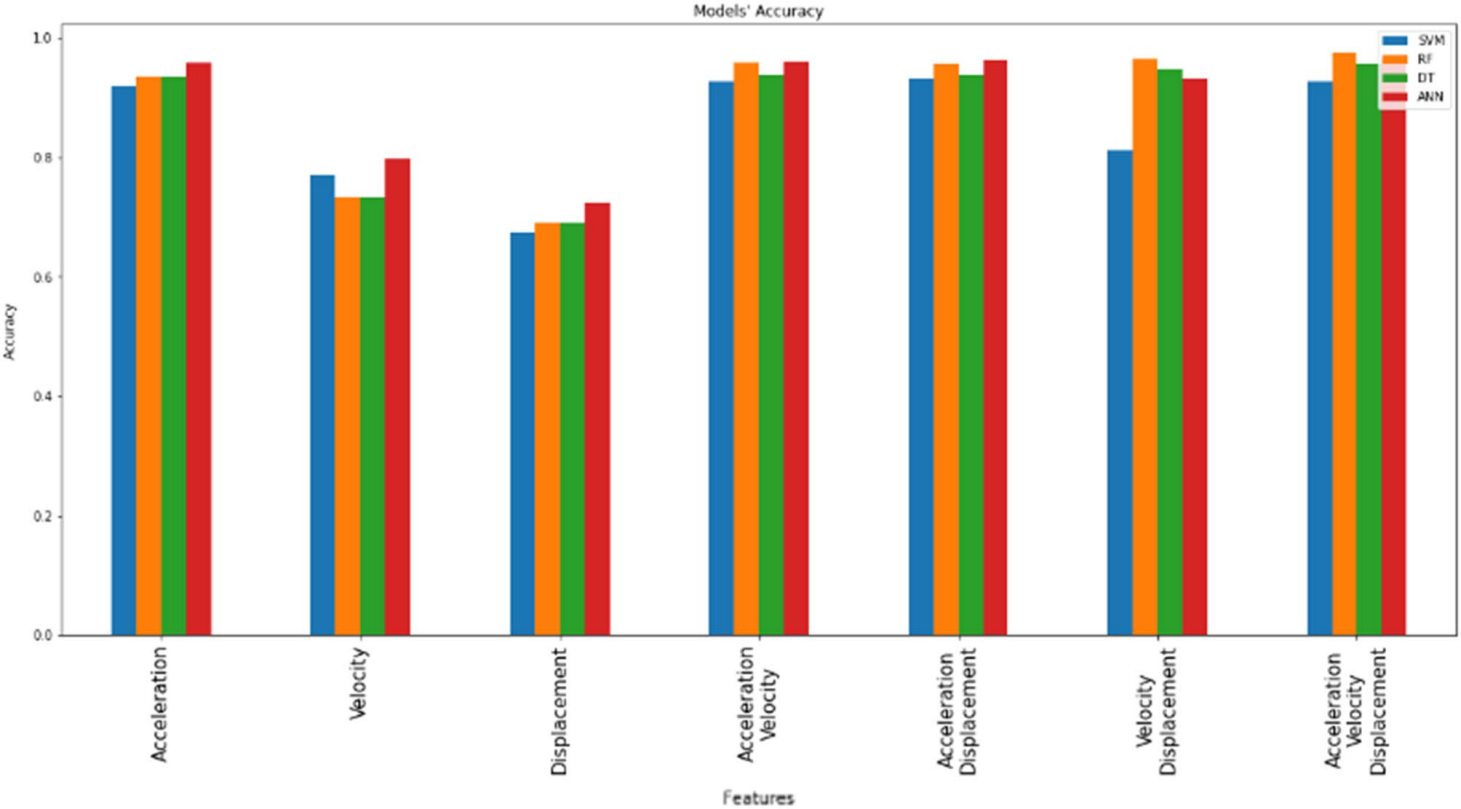
Models’ accuracy chart.

Analysis of model performance is carried out by comparing the values for accuracy, precision, recall, and F1 on each algorithm. Based on the experimental data in Table 3, the accuracy is in the range of 0.673230 – 0.965400, precision is in the range of 0.656123 – 0.974964, recall is in the range of 0.673230 – 0.974589, and F1 is in the range of 0.65009 – 0.97458. The two highest values of the models in all possibilities of the input features are dominated by ANN followed by RF but ANN shows better resistance to various input features than RF. The experiment shows that with acceleration as a feature, all models' performances look good, but when velocity and displacement stand alone as a feature, every model has low performance. However, when velocity and displacement are used together as a feature, all models’ performances increase significantly. In addition, all possibilities of the input features were used in this experiment. Combining acceleration with velocity and acceleration with displacement has been proven to improve the performances of models based on experimental data in Table 3 as can be seen in Fig. 8. Furthermore, 3 out of 4 models’ performances show improvement when 3 of them are used as a feature, only SVM shows a decrease in performance. It can be seen from Fig. 8, the best SVM performance for using a single feature was obtained when acceleration was used. Followed by displacement and velocity. This is due to the fact that in case of adding more feature data that is less sensitive to the desired output, a redundancy in the input data might lead to occur. As a result, the model might translate the additional information as false information that leads to difficulty in detecting the real pattern of the data and therefore achieving low accuracy. Therefore, it can be concluded that only the highly correlated and important feature is acceleration. And that can be confirmed when acceleration was used as one of two or three features. Therefore, it can be concluded that adding velocity and displacement as additional features can improve models’ performances. Hence, in order to achieve a high level of accuracy, acceleration must be included as a feature of each model for better earthquake detection.

**Table 3:** Machine learning performances.

Features	Algorithm	Confusion matrix	Accuracy	Precision	Recall	F1
Acceleration	SVM	$$\left[ {\begin{array}{*{20}c} {3460} & 0 & 0 & 0 \\ 0 & {2815} & {656} & 0 \\ 0 & 1 & {3504} & 0 \\ {148} & {69} & 0 & {12} \\ \end{array} } \right]$$	0.918049	0.926970	0.918049	0.90852
	RF	$$\left[ {\begin{array}{*{20}c} {3460} & 0 & 0 & 0 \\ 0 & {3128} & {316} & {26} \\ 0 & {309} & {3196} & 0 \\ 0 & {27} & 0 & {202} \\ \end{array} } \right]$$	0.936333	0.936329	0.936333	0.93633
	DT	$$\left[ {\begin{array}{*{20}c} {3460} & 0 & 0 & 0 \\ 0 & {3129} & {315} & {27} \\ 0 & {321} & {3184} & 0 \\ 0 & {27} & 0 & {202} \\ \end{array} } \right]$$	0.935302	0.935308	0.935302	0.93530
	ANNs	$$\left[ {\begin{array}{*{20}c} {3460} & 0 & 0 & 0 \\ 0 & {3123} & {339} & 0 \\ 0 & {48} & {3457} & 0 \\ {26} & {29} & 0 & {174} \\ \end{array} } \right]$$	0.958556	0.960421	0.958556	0.95816
Velocity	SVM	$$\left[ {\begin{array}{*{20}c} {3460} & 0 & 0 & 0 \\ {309} & {2331} & {831} & 0 \\ {79} & {1011} & {2415} & 0 \\ {13} & {129} & {87} & 0 \\ \end{array} } \right]$$	0.769432	0.747423	0.769432	0.75735
	RF	$$\left[ {\begin{array}{*{20}c} {3453} & 7 & 0 & 0 \\ {12} & {2162} & {1201} & {96} \\ 0 & {1217} & {2185} & {103} \\ 0 & {119} & {105} & 5 \\ \end{array} } \right]$$	0.731833	0.730278	0.731833	0.73104
	DT	$$\left[ {\begin{array}{*{20}c} {3453} & 7 & 0 & 0 \\ {13} & {2171} & {1192} & {95} \\ 0 & {1228} & {2174} & {103} \\ 0 & {120} & {104} & 5 \\ \end{array} } \right]$$	0.731645	0.730025	0.731645	0.73081
	ANNs	$$\left[ {\begin{array}{*{20}c} {3453} & 7 & 0 & 0 \\ {13} & {2171} & {1192} & {95} \\ 0 & {1228} & {2174} & {103} \\ 0 & {120} & {104} & 5 \\ \end{array} } \right]$$	0.798218	0.783111	0.798218	0.79028
Displacement	SVM	$$\left[ {\begin{array}{*{20}c} {3460} & 0 & 0 & 0 \\ {749} & {2109} & {613} & 0 \\ {450} & {1444} & {1611} & 0 \\ {12} & {137} & {80} & 0 \\ \end{array} } \right]$$	0.673230	0.656123	0.673230	0.65009
	RF	$$\left[ {\begin{array}{*{20}c} {3380} & {53} & {27} & 0 \\ {136} & {1886} & {1348} & {101} \\ {29} & {1318} & {2082} & {76} \\ 0 & {126} & {77} & {26} \\ \end{array} } \right]$$	0.691420	0.687131	0.691420	0.68918
	DT	$$\left[ {\begin{array}{*{20}c} {3409} & {33} & {18} & 0 \\ {152} & {1932} & {1287} & {100} \\ {49} & {1375} & {2005} & {76} \\ 0 & {128} & {75} & {26} \\ \end{array} } \right]$$	0.691233	0.685098	0.691233	0.68795
	ANNs	$$\left[ {\begin{array}{*{20}c} {3460} & 0 & 0 & 0 \\ {222} & {2471} & {778} & 0 \\ {109} & {1596} & {1800} & 0 \\ 0 & {143} & {86} & 0 \\ \end{array} } \right]$$	0.724894	0.709179	0.724894	0.71080
Acceleration & velocity	SVM	$$\left[ {\begin{array}{*{20}c} {3460} & 0 & 0 & 0 \\ 0 & {2786} & {685} & 0 \\ 0 & 0 & {3505} & 0 \\ {36} & {49} & 0 & {144} \\ \end{array} } \right]$$	0.927801	0.937305	0.927801	0.92629
	RF	$$\left[ {\begin{array}{*{20}c} {3460} & 0 & 0 & 0 \\ 0 & {3169} & {301} & 1 \\ 0 & {128} & {3377} & 0 \\ 0 & {21} & 0 & {208} \\ \end{array} } \right]$$	0.957712	0.958386	0.957712	0.95767
	DT	$$\left[ {\begin{array}{*{20}c} {3460} & 0 & 0 & 0 \\ 0 & {3149} & {305} & {17} \\ 0 & {314} & {3191} & 0 \\ 0 & {21} & 0 & {208} \\ \end{array} } \right]$$	0.938396	0.938411	0.938396	0.93840
	ANNs	$$\left[ {\begin{array}{*{20}c} {3460} & 0 & 0 & 0 \\ 0 & {3151} & {319} & 1 \\ 0 & {71} & {3434} & 0 \\ 6 & {24} & 0 & {199} \\ \end{array} } \right]$$	0.960525	0.961871	0.960525	0.96040
Acceleration & displacement	SVM	$$\left[ {\begin{array}{*{20}c} {3460} & 0 & 0 & 0 \\ 0 & {2810} & {661} & 0 \\ 0 & 0 & {3505} & 0 \\ {44} & {34} & 0 & {151} \\ \end{array} } \right]$$	0.930707	0.939890	0.930707	0.92940
	RF	$$\left[ {\begin{array}{*{20}c} {3460} & 0 & 0 & 0 \\ 0 & {3156} & {314} & 1 \\ 0 & {135} & {3370} & 0 \\ 0 & {11} & 0 & {218} \\ \end{array} } \right]$$	0.956774	0.957500	0.956774	0.95674
	DT	$$\left[ {\begin{array}{*{20}c} {3460} & 0 & 0 & 0 \\ 0 & {3127} & {335} & 0 \\ 0 & {310} & {3195} & 0 \\ 0 & {10} & 0 & {219} \\ \end{array} } \right]$$	0.937740	0.937750	0.937740	0.93773
	ANNs	$$\left[ {\begin{array}{*{20}c} {3460} & 0 & 0 & 0 \\ 0 & {3135} & {336} & 0 \\ 0 & {39} & {3466} & 0 \\ 5 & {13} & 0 & {211} \\ \end{array} } \right]$$	0.963150	0.965177	0.963150	0.96305
Velocity & displacement	SVM	$$\left[ {\begin{array}{*{20}c} {3460} & 0 & 0 & 0 \\ {319} & {2239} & {913} & 0 \\ {95} & {591} & {2819} & 0 \\ {13} & {64} & 0 & {152} \\ \end{array} } \right]$$	0.812939	0.810299	0.812939	0.80770
	RF	$$\left[ {\begin{array}{*{20}c} {3460} & 0 & 0 & 0 \\ 0 & {3163} & {308} & 1 \\ 0 & {66} & {3439} & 0 \\ 0 & 3 & 0 & {226} \\ \end{array} } \right]$$	0.964650	0.966037	0.964650	0.96460
	DT	$$\left[ {\begin{array}{*{20}c} {3460} & 0 & 0 & 0 \\ 1 & {3149} & {318} & 3 \\ 0 & {239} & {3266} & 0 \\ 0 & 5 & 0 & {224} \\ \end{array} } \right]$$	0.946929	0.947058	0.946929	0.94691
	ANNs	$$\left[ {\begin{array}{*{20}c} {3460} & 0 & 0 & 0 \\ {80} & {2959} & {432} & 0 \\ 0 & {204} & {3301} & 0 \\ 4 & {12} & 0 & {213} \\ \end{array} } \right]$$	0.931364	0.932136	0.931364	0.93080
Acceleration & velocity & displacement	SVM	$$\left[ {\begin{array}{*{20}c} {3460} & 0 & 0 & 0 \\ 0 & {2776} & {695} & 0 \\ 0 & 0 & {3305} & 0 \\ {37} & {37} & 0 & {155} \\ \end{array} } \right]$$	0.927894	0.937903	0.927894	0.92658
	RF	$$\left[ {\begin{array}{*{20}c} {3460} & 0 & 0 & 0 \\ 0 & {3277} & {194} & 0 \\ 0 & {69} & {3436} & 0 \\ 0 & 8 & 0 & {221} \\ \end{array} } \right]$$	0.974589	0.974964	0.974589	0.97458
	DT	$$\left[ {\begin{array}{*{20}c} {3460} & 0 & 0 & 0 \\ 0 & {3243} & {225} & 3 \\ 0 & {240} & {3265} & 0 \\ 0 & 8 & 0 & {221} \\ \end{array} } \right]$$	0.955368	0.955404	0.955368	0.95537
	ANNs	$$\left[ {\begin{array}{*{20}c} {3460} & 0 & 0 & 0 \\ 0 & {3156} & {314} & 1 \\ 0 & {39} & {3466} & 0 \\ 2 & {13} & 0 & {214} \\ \end{array} } \right]$$	0.965400	0.967137	0.965400	0.96532

## Conclusion

This research has focused on addressing the multi-classification of the earthquake and non-earthquake vibrations through the application of machine learning. Three different widely adopted algorithms were developed, namely: Support Vector Machine (SVM), Random Forest (RF), Decision Tree (DT), and Artificial Neural Network (ANN). These models have been utilized in vibration multi-classification detection namely earthquake, non-earthquake, made-up earthquake, and heavy vehicles passing by. The results of the models were evaluated by four different performance criteria: accuracy, precision, recall, and F1 score. Comparing those performances criteria of SVM, RF, DT, and ANN, this study concludes that ANN outperforms other machine learning algorithms in 6 out of 7 possibilities of the input features. In addition, ANN shows better resistance to various input features. Hence, ANN has been proposed as the best algorithm which can be used for multi-classification earthquake detection based on this experiment. Furthermore, acceleration, velocity, and displacement show a good correlation with the result that combining acceleration, velocity, and displacement has been proven can be used to improve the model’s performances. Summing up, combining those features improves the accuracy of RF and ANN models respectively to 0.974589 and 0.965400. for future research, future studies should be made on multi-classification earthquake detection using ANN specifically and implement it in hardware, to further prove its capability in multi-classification earthquake detection in real-time. Despite having an acceptable accuracy value to classify earthquake detection, flaws and limitation remain. There is a need to restructure the model to achieve the best possible and optimal architecture model so that the model can distinguish vibration more accurately. And the reliability of the proposed models in the future might be validated by using more available data.

## Data Availability

The datasets generated during the current study are not publicly available which is considered as copyright between the funded agency and the university but are available from the corresponding author on reasonable request.
